# Integrating technology into complex intervention trial processes: a case study

**DOI:** 10.1186/s13063-016-1674-9

**Published:** 2016-11-17

**Authors:** Cheney J. G. Drew, Vincent Poile, Rob Trubey, Gareth Watson, Mark Kelson, Julia Townson, Anne Rosser, Kerenza Hood, Lori Quinn, Monica Busse

**Affiliations:** 1 South East Wales Trials Unit, Centre For Trials Research, College of Biomedical and Life Sciences, Cardiff University, 7th Floor Neuadd Meirionnydd, Heath Park, Cardiff, CF14 4YS UK; 2 School of Biosciences, Cardiff University, Biosciences 3, Park Place, Cardiff, CF10 3BB UK; 3 Department of Biobehavioral Sciences, Teachers College, Columbia University, New York, NY USA; 4 School of Health Care Sciences, Cardiff University, 35-43 Eastgate House, Newport Road, Cardiff, CF24 0AB UK

**Keywords:** Technology, Data collection, Complex intervention trials, Database design, Fidelity, Process evaluation

## Abstract

**Background:**

Trials of complex interventions are associated with high costs and burdens in terms of paperwork, management, data collection, validation, and intervention fidelity assessment occurring across multiple sites. Traditional data collection methods rely on paper-based forms, where processing can be time-consuming and error rates high. Electronic source data collection can potentially address many of these inefficiencies, but has not routinely been used in complex intervention trials. Here we present the use of an on-line system for managing all aspects of data handling and for the monitoring of trial processes in a multicentre trial of a complex intervention.

We custom built a web-accessible software application for the delivery of ENGAGE-HD, a multicentre trial of a complex physical therapy intervention. The software incorporated functionality for participant randomisation, data collection and assessment of intervention fidelity. It was accessible to multiple users with differing levels of access depending on required usage or to maintain blinding. Each site was supplied with a 4G-enabled iPad for accessing the system. The impact of this system was *quantified* through review of data quality and *collation of feedback* from site coordinators and assessors through *structured process* interviews.

**Results:**

The custom-built system was an efficient tool for collecting data and managing trial processes. Although the set-up time required was significant, using the system resulted in an overall data completion rate of 98.5% with a data query rate of 0.1%, the majority of which were resolved in under a week. Feedback from research staff indicated that the system was highly acceptable for use in a research environment. This was a reflection of the portability and accessibility of the system when using the iPad and its usefulness in aiding accurate data collection, intervention fidelity and general administration.

**Conclusions:**

A combination of commercially available hardware and a bespoke online database designed to support data collection, intervention fidelity and trial progress provides a viable option for streamlining trial processes in a multicentre complex intervention trial. There is scope to further extend the system to cater for larger trials and add further functionality such as automatic reporting facilities and participant management support.

**Trial registration:**

ISRCTN65378754, registered on 13 March 2014.

## Background

Running a multicentre complex intervention trial generates significant trial management demands both centrally and in local teams independent of the complexity of the trial design. Complex intervention trials are likely to require data collection via multiple formats in order to monitor and record multiple and differing aspects of the intervention [1]. These may include the more traditional paper Case Report Forms (CRFs), video observations and audio-recordings, the latter usually requiring multiple pieces of specialist equipment, all contributing to increased costs. Complex interventions also require some degree of assessment of how accurately the intervention is delivered to ensure consistency of delivery across multiple sites. Again this can be costly in terms of personnel hours required and can contribute to the general inefficiency of a trial.

Paper CRFs remain the most widely used data collection tool and are perceived to be quick to implement and relatively simple to manage [2]. Benefits of using paper CRFs include the ease of testing, distribution to sites and simplicity at the point of study closure and archiving. However, the need for transcription to electronic systems and/or photocopying of paper CRFs for sending to the trial centre is costly and introduces likely avenues for error and duplication of effort [3]. Further, lengthy monitoring visits may be required to ensure the quality and appropriateness of the data being collected, necessitating comparison of transcribed records against the original source material. A move to electronic source CRF (eSource) data collection provides a method for reducing this burden. Initial set-up can be time-consuming, however, and additional training is often required for correct usage [3]. Currently, eSource data collection has failed to replace traditional paper CRFs in most complex intervention trial environments, although the use of web-accessible data capture methods to improve trial efficiency is an area of emerging interest.

Our aim was to use modern and portable eSource technologies to improve many of the management and data collection errors and inefficiencies that are often experienced in complex intervention trials, and to test this in a small-scale study. ENGAGE-HD is a multicentre, single-blinded complex intervention trial of a complex physical activity intervention in Huntington’s disease where the intervention was delivered at the participant’s home. Trial assessments were performed over three time points at eight sites across the UK [4] Although a relatively small trial, there were a number of specific complexities that made this a good case study candidate for establishing proof of principle for such a system: each site had a coordinator, one or more coaches for the physical and social (control) intervention and assessors blinded to the arm allocation of the participant. Briefly, site coordinators scheduled appointments and visits, aided data collection and acted as the main point of contact for the Central Study Team. The blinded assessors conducted the evaluations for the primary trial outcomes at three time points in a hospital-based setting, with each assessment requiring the completion of 9–15 individual CRFs. The intervention coaches visited participants at their home on six occasions between assessments 1 and 2 where they were required to deliver the intervention and collect weekly diary data from participants. Therefore, data collection needed to be as efficient as possible to maximise data capture across all settings and time points. In this paper we describe the system development, implementation and methods to capture impact of a custom-designed web-based system developed with the intention of streamlining trial processes in ENGAGE-HD.

## Methods

### System development

The web-based system for ENGAGE-HD was designed to be a robust, intuitive and flexible system with the primary aim of aiding trial management and data collection. We incorporated a number of differing functional modules into the system which provided a facility for monitoring and assessing intervention fidelity and for performing the randomisation of participants. Development took a period of 3 months, encompassing alpha-testing, which focussed on the initial look and feel of the system, and beta-testing which focussed on the user acceptability of the system. Development and testing was undertaken by an in-house software developer at an approximate cost of £7250, with data manager input at an approximate cost of approximately £9134 for the initial development. We estimate that the developer provided maintenance and ongoing support for 3 h over the duration of the recruitment period which was an additional cost of £3782. This culminated in primarily eSource data collection, with the option of using paper CRFs where desired or required. All system users were issued with their own username and password to access the facility with differing levels of permissions conferred to individual users dependent on their role. For example, the blinding of assessors was maintained by denying them access to participant allocation information and the intervention coaches were given specific access to upload audio-recordings to the database and to access a summary report of the subsequent fidelity evaluation.

The platform was created to be accessible through desktop computers and tablet devices to allow for maximum functionality and real-time data capture. However, where site staff felt more comfortable with completing paper CRFs, the software was designed so that the CRF could be generated as a PDF directly from the platform and printed for manual use. The software was also built to enable participant randomisation after the completion of the initial assessment without the need to contact the Central Study Team and also allowed for real-time data entry during home visits and assessments. Randomisation was performed using minimisation [5] in order to balance between groups based on data obtained at the baseline assessment (age, sex and Unified Huntington’s Disease Rating Scale-Total Motor Score (UHDRS-TMS).

The software added extra functionality as a data collection tool with in-built data validation rules to minimise errors at the point of data entry. With controllable format of data entry fields, data could only be entered in the correct format, and values outside of prespecified ranges generated a warning message to alert the researcher before the form was saved. The system was also engineered with the capability to upload audio-recordings for fidelity assessment. Intervention coaches could record the audio of their interaction with the participant using the iPad, which was then securely uploaded to the database before being deleted according to data protection strictures. The audio-recording was rated for fidelity of the delivery of the intervention in line with the theoretical framework on which it was developed [6]. Feedback from the intervention trainer was delivered to intervention coaches via Skype® on the tablet device.

The software design automatically stored inputted data in a structured query language (SQL) database from which interim extracts could be made available on demand. Further design aspects enabled the Central Study Team to receive e-mail alerts once assessment data had been submitted so that data queries could be raised and resolved in a timely fashion. Any changes made to the data forms after the original submission were automatically saved, logging all details required by Good Clinical Practice (GCP) requirements: the user who made the change, the date of the change and the values before and after the change.

### Device configuration

The web-based system was intended to be accessible to researchers in real time so we decided to invest in portable, web-enabled devices to make this possible. A number of factors were considered in the choice of device to be used in the trial. These ranged from availability, cost, portability and security to the ease and acceptability of use in the trial setting. We concluded that the device which would offer the best compromise over all was the iPad Air® as it is a widely used device, with uniform interface design across most applications and would require the least amount of additional training for users. The ability to use the video-conferencing software Skype® and integrated video- and audio-recording meant that a single device had all the required functionality to perform all trial-related tasks at site. All devices were configured to use a secure Wi-Fi connection where available or to fall back on 4G data services, which allowed reliable connectivity at all research sites.

### Implementation

Prior to implementation at research sites, the system was fully tested and validated using all devices and interfaces that would be used as part of the trial. Each site was visited by the Central Study Team for staff training in using the iPad, uploading data through the website and the use of the supplementary software packages. Checks were also carried out on the availability of secure Wi-Fi access and cellular network coverage. Each site was required to provide their agreement to use the device and system as instructed and to maintain device updates where necessary. Support and additional training was also given through e-mail and telephone contact with the trial manager on a needs basis.

#### Methods to capture impact

Our evaluation of the impact of the system was based on two principal criteria: (1) trial data quality and (2) user feedback. To assess data quality, we made a comparison of the number of data queries raised to the number of data items entered and the time taken to resolve data queries. We also descriptively summarised the proportion of missing data items in the minimum dataset. We also noted how many sites were using paper CRFs by the third and final participant assessment.

Feedback was obtained through structured telephone interviews with members of research staff from across six of the eight sites involved in the trial. We aimed to get feedback from at least one person per site who had used the database. We were able to conduct interviews with six members of site staff which included: two intervention coaches, three site coordinators (one of whom also acted as a coach) and one blinded assessor. Interviews consisted of a predetermined set of ten questions covering topics such as the usability and accessibility of the system and support requirements. Interviews were conducted by the database developer and responses to questions were recorded verbatim in real time. Responses were summarised according to the questions asked using the method of qualitative description [7, 8].

#### Trial participants

Target recruitment for ENGAGE-HD inflated for losses to follow-up was 62, requiring 46 for final analysis. Forty-six participants were recruited (*n* = 25 male and *n* = 21 female). Mean age was 59.4 years (standard deviation (SD) 10.1).

## Results

### Training and on-site support

The development of our bespoke, purpose-built electronic trial management system required intensive resource in the initial set-up period. Significant time was needed to accurately write all the necessary metadata, which included the use of in-built validation rules to prevent the entry of erroneous data. Although not formally recorded, we estimate that the development of the database required 12 weeks of data manager time and approximately 10 weeks of developer time, running concurrently, to build and sufficiently test the system.

The initial site set-up process required intensive training during the initiation visit. In addition to standard site initiation tasks, an extra 1–2 h was set aside to familiarise key staff with the use of the iPad and the functions of the ENGAGE-HD database. The requirement for additional training differed between individual sites, based largely on staff familiarity with using iPads or other electronic tablet devices. Step-by-step guides were produced to help staff with the use of additional applications required for audio- and video-recording. Further support (via e-mails and telephone calls) was also sometimes required during the initial months after recruitment began until staff were sufficiently confident with using the system.

### Data quality

We evaluated the impact of the system on data collection and quality by looking at the completeness and quality of the data that was received through the database. Table 1 shows the number of completed electronic CRFs broken down by individual study sites. Two sites achieved a completion rate of 100%, with the site returning the fewest completed forms still achieving a completion rate of 96%. Across the study, 2639 of 2678 CRFs were successfully completed, giving an overall return rate of 98.5%. Individual sites were contacted for missing CRFs on multiple occasions and this was monitored monthly by the Trial Management Group. After a period of approximately 3 months, as none of the data contained in the missing CRFs were critical for the primary trial analysis, the Trial Management Committee decided to record this 1.5% of nonreturned CRFs as missing data.

**Table 1:** Completion of electronic Case Report Forms (CRFs) and the number of data queries by study site

Study site	Number of participants recruited	Number of required CRFs	Number of completed CRFs	Completed CRFs (% total)	Number of Data queries	Median time to query resolution (days)
1	10	654	643	98.3	22	4
2	2	124	120	96.8	6	1.5
3	4	239	237	99.2	22	1
4	5	325	325	100.0	18	1.5
5	6	337	337	100.0	22	6
6	4	241	240	99.6	8	19
7	10	576	563	97.7	18	2
8	5	182	174	96.0	25	2
Total	46	2678	2639	98.5	141	3

We were also able to assess the number of data queries per site and the median time it took to resolve those data issues. Overall there were 141 data queries for the whole trial, which were resolved in a median time of 3 days (Table 1). All but one site routinely resolved data queries within 1 week of the query being raised. We calculated that for participants in the physical intervention there would be a total of 3134 data points across the whole trial and 1674 for participants in the social arm. Assuming that all participants completed the trial as intended and that all CRFs were completed, this would give a total of over 100,000 individual data points. Therefore, the number of data queries raised across the trial constitutes approximately 0.1% of all the data entered for the trial.

In order to assess fidelity, coaches in the intervention arm were also asked to audio-record one of their home visits, using an in-built iPad application and then upload that file to the Central Study Team through the ENGAGE-HD database. Sixteen participants completed the intervention, and in all cases the coaches successfully uploaded an audio-recording via the ENGAGE-HD database. Audio-recordings captured by the iPad device were of sufficient sound quality to be accurately transcribed, and formed the basis of further fidelity analysis reported elsewhere [6].

Lastly, we reviewed the reported protocol deviations for the trial to assess the impact of the database on protocol adherence. Out of the 30 protocol deviations reported for the ENGAGE-HD trial, three were related to the use of the database. Two incidents could be attributed to user error: the wrong age category was selected on the randomisation form, and one audio-recording was not uploaded to the database within 48 h of being taken so it could be deleted from the iPad. The other incident was a case where the blinded assessor had difficulty accessing the database and was unable to upload the baseline information immediately. Although the data was recorded on back-up paper copies, the process lead to a delay in the randomisation of the participant so that they were not informed of their group allocation until a number of days after the assessment. The other incidences of protocol deviations were largely attributed to scheduling issues or intrasite communications leading to the unblinding of assessors.

### User impact

We considered the impact of the integrated system on both the trial staff and the site staff. For trial staff, one of the major advantages we found with using this system was that it allowed real-time monitoring of the progress of the trial and of individual participants. The database was programmed to send e-mail alerts to the trial and data managers as soon as participant assessment data had been entered. This enabled prompt review of the data collected, allowing swift generation and resolution of data queries, as described above. Further, this level of access allowed the Central Study Team to monitor the progress of intervention visits and flag up if there were any obvious delays. If such a problem was noted, communication with the site could be initiated immediately to offer further support and advice if required.

We performed semi-structured interviews with a mix of site staff (*n* = 6) involved in ENGAGE-HD to investigate their views on the impact of the database system on the delivery of the study. A summary of the results from these interviews can been in Table 2.

**Table 2:** Summary of feedback responses given by ENGAGE-HD research staff in semistructured interviews

Question	Response		Number of respondents
Have you previously collected research data for studies? If yes, how did you collect the data?	No		1
	Yes, mainly using paper Case Report Forms (CRFs)		1
	Yes, using paper CRFs then entered into a database		4
What was your experience of using an iPad prior to your work on ENGAGE-HD?	None		1
	Limited		2
	Personal use		4
What was your experience of using an iPad on ENGAGE-HD?	Positive		6
What were some of the advantages?	Mobility/ability to be able to work across NHS and other sites.		4
	Ease of use		5
	Facilitation of data entry		3
	3G/Signal		2
	In-built data validations		1
	Ability to use additional apps		3
What were some of the disadvantages?	None		1
	System crashed once		1
	Repeated log-ons (at each visit to the database web page) slowed process		1
	Only one device per site		1
	Form design not optimum (for giving feedback and because of repetitive data entry).		2
	Poor battery life		1
Did you use the added functionality of the iPad during the trial? If so how?	No		0
	Yes	Skype®	5
		Camera for photographing documents	2
		Audio-recording	4
		Secure e-mail	2
Was the support you were given adequate?	No		0
	Yes		6
Has the use of the iPad on ENGAGE-HD influenced your view on working with it in clinical trials in future?	Not really		1
	Yes	Would use in future studies	3
		A useful option	1
		But might not work for all studies such as text-heavy qualitative studies, etc.	1

One interviewee was new to the collection of trial data, but the remainder had performed similar work before and they all indicated that the majority of data collection was using traditional paper CRFs. Three people mentioned that data collected on paper CRFs was then entered on to a database via a desktop PC and a further person said that they had worked on studies previously where data was uploaded directly into a database.

Half of the staff interviewed indicated that their experience of using iPads was limited and although the remainder had experience through personal use, no one had previously used an iPad in a work context.

All respondents were extremely positive about the training they received for using the iPad and database. All respondents stated that the training and support they received was adequate and no one had any suggestions for improvements. Specific responses highlighted that queries were responded to quickly, the standard operating procedures (SOPs) provided were clear and easy to follow and that the site initiation training was good. Half of the staff interviewed (*n* = 3) also singled out that efficient management of the trial was important in how well supported they felt.

To gain more specific perspectives of site staff, we asked them to describe the relative advantages and disadvantages of fully eSource data collection using the supplied iPad. The summary of responses can be found in Table 2. These included benefits such as ease of use and data collection, mobility of use and the ability to use the additional apps to facilitate the work. Some of the drawbacks mentioned included limited battery life, only having one device per site, and that on-line forms could be redesigned to allow more feedback.

All interviewees said that they took advantage of some the additionally functionality (Skype®, audio-recording, camera, e-mail) of using an iPad. The specific apps and the amount they were used were dependent on the research role of the specific interviewee. Intervention coaches particularly liked the use of the audio-recording and Skype® functions to discuss and receive feedback on the delivery of the intervention with the intervention trainer. Site coordinators employed the use of the camera to take high-resolution images of documents which could then be e-mailed securely to the coordinating centre.

Lastly, we asked the site staff if their experience of using the iPad and database for ENGAGE-HD had influenced their views on working on trials in the future. The response to this was overwhelmingly positive, with the move to paperless systems being particularly popular, although it was noted that different studies would have specific requirements.

Overall the feedback of the research staff interviewed on the use of the iPad and database for delivering ENGAGE-HD demonstrated high levels of acceptability to site users.

## Discussion

Through this case study, we have demonstrated that is possible to produce reliable, web-accessible software for the purposes of data collection and management and trial management in a multisite trial of a complex intervention. The return rate and quality of data collected was particularly high and the system had high user acceptability ratings. The use of the system also allowed for efficient trial management by enabling sites to perform randomisation of participants themselves and by reducing the need for extensive and repeated on-site data monitoring, which offset the initial intensive training required during site set-up.

The system we designed facilitated the immediate collection of source data during study visits and assessments via use of the iPad, negating the need for secondary data transcription once it had been collected. It seems likely that this portable collection facility, which incorporated prompts and validation checks in real time, ensured that the overall form completion rate for the trial was high. Importantly, the software enabled the facility to provide paper back-ups of all CRFs when access to the live system was not possible or desired and, as such, data collection was not compromised.

Data validation rules that were embedded into the software ensured that the accuracy of completed eSource data was high, resulting in a low administrative burden in terms of generating and resolving data queries. This specific database design feature allowed us to be confident that when assessment data had been entered and checked the data was sufficiently clean for analysis, thereby negating the need for lengthy data cleaning once the trial was finished. We believe that this time-saving efficiency more than compensated for the higher additional set-up time required for developing and programming the software. Additionally, the combination of high levels of data completion and accuracy along with the efficient site communication meant that intensive site-monitoring visits were not deemed necessary to ensure the efficient delivery of the trial.

The lack of multiple copies of paper CRFs in this trial also conferred other benefits. Firstly, archiving at sites can be problematic due to constraints on space in suitable facilities. The reduction in the volume of paperwork generated in ENGAGE-HD through the implementation of a largely paperless system is, therefore, an important consideration. Secondly, we were able to reduce the number of costly site-monitoring visits, due to a decreased need to carry out quality control inspections of paper records against the transcribed copies on digital systems.

The use of this web-based system in ENGAGE-HD had added benefits beyond data collection. The randomisation was embedded in the programme ensuring that participants could be effectively randomised on the day of their screening assessment without the need for contacting the coordinating centre. This feature reduced the timelines of progressing any given participant through the trial by removing the need for staff at the sites and the coordinating centre to communicate directly. Remote and automated randomisation in itself is not novel and the approach has been published elsewhere [9–11] as an effective method for allocating participants, but to our knowledge it has not previously been embedded within the study data collection system alongside other trial-monitoring and management features. Perhaps, the most important design feature of the ENGAGE-HD database was the integrated method for fidelity monitoring, a vital aspect of delivering complex interventions. This allowed rapid assessment of the fidelity of the intervention delivery by all coaches with all participants in the physical intervention arm and for useful feedback to be delivered in a useful and timely fashion. For all participants in the intervention arm, an audio-recording was successfully uploaded for fidelity assessment, which indicates the utility of the database for this process. Additionally, the individualised permissions’ settings designed for each user enabled intervention coaches to access confidential feedback on their delivery of the intervention through the system, which they could refer back to at any time. We believe that this aspect of the system was key to the high levels of intervention fidelity in the trial which we have reported elsewhere [6].

It was important to gain the perspectives of end users of the database who were not involved in its development to determine the acceptability of the technology in a research environment. If end users are not comfortable or satisfied with the data collection and management modules, they will either not engage with using the system or, if enforced, their use maybe more inefficient and less accurate. In general, the feedback we received from site staff through the telephone interviews conducted was positive and the move from paper CRF data collection to eSource was widely welcomed. Intervention coaches were particularly receptive to the use of Skype® and audio-recordings for fidelity monitoring and found the feedback received useful for intervention delivery [6].

Some staff had reservations about using the iPad and navigating the database whilst assessing participants, but we found that additional support and training from the coordinating centre alleviated these fears. Further, the feedback that we received revealed that the additional benefit of using an iPad on a cellular network was in part that it was unaffected by many of the security features present on NHS networks, which prevent the use of some web-enable applications such as Skype®.

This case study provides preliminary indications that our system was indeed beneficial to trial processes. We do, however, acknowledge that our evaluation of impact could have been strengthened by obtaining a wider range of perspectives. A better approach would have been to conduct face-to-face interviews with site staff rather than rely on a structured telephone interview.

We note that a major limitation in the evaluation of the system as a whole is the lack of comparator (such as a purely paper-based system), which is necessary to draw firm conclusions about the efficiency of our system. ENGAGE-HD was a feasibility trial of a complex intervention and we did not plan to formally evaluate the efficacy of our system in reducing data queries or data cleaning. In spite of differential data query rates across our sites, the overall data return rate across all ENGAGE-HD sites was, however, similar. Furthermore the real-time monitoring of data and in-built data validations to our system meant that data cleaning occurred automatically. This suggests that the system described here provides the added advantage of site support during data collection but we are not able to confirm this without a full-scale comparative evaluation. We await, with interest, the results of the TRANSFoRM study which is currently evaluating the utility of an electronic platform in the recruitment and follow-up of participants in primary care research [10] as this is likely to provide more support for the approach we describe here.

Whilst we found that the use of the on-line database and portable technologies in the delivery of ENGAGE-HD were largely beneficial, we recognise that this system had limitations. In order for the system to be functional, internet access is required at all times; therefore, secure Wi-Fi or adequate cellular coverage must be available. We circumnavigated this issue by purchasing 4G-enabled tablet devices, but recognise that this adds a significant extra cost. Although lack of accessibility was an initial concern, we resolved this by ensuring that paper copies could be obtained without internet access. This ensured that accessibility did not become an issue during the trial.

One of the aims of the ENGAGE-HD trial was to design and implement an on-line system to streamline processes associated with fidelity monitoring, participant randomisation, site communication and data collection in a multicentre complex intervention trial. Electronic data capture is becoming increasingly popular as a method of data acquisition in clinical trials for reducing both the time and costs required to deliver a study [12, 13], but the number of studies using such applications remains comparatively small [14]. Current methods of electronic data capture usually require the transcription of paper records to eSource via manual input or scanning technologies, both of which can introduce error and a significant time burden on the research team. With both of these methods, data still needs to be checked for accuracy and validity, further adding to trial running costs. Such processes contribute to general inefficiencies in trials where large amounts of data are being collected [15]. Additional time-saving benefits of automated trial processes and data collection compared to traditional paper CRF methods and management are being increasingly recognised in industry-sponsored trials [12, 16, 17].

For future versions of a similar trial database, we would like to investigate the addition of further functional modules that could further reduce time burdens in administration and recording of necessary documentation. For example, we found that although protocol deviations were reported in a timely fashion, there were significant delays in receiving the necessary signed documentation. We believe that by transferring this process to an on-line facility, documentation would be completed in a more timely fashion. Similarly, we would integrate screening logs and safety reporting into future versions of the database to improve the speed of documentation return and improve consistency between sites.

In this trial we continued to monitor outcome data as it was uploaded to check accuracy and completeness. As a result, future iterations should take further time during the set-up period to add-in additional, stricter validation rules or ‘flags’ for specific outcomes. A flagging system could be incorporated so that data outside of accepted normative values could produce a prompt to check the validity of the data entered. Coupled with ‘free-text’ sections, where those entering data could add further detail and explanation, this would prevent largely erroneous data from being entered and reduce the need for ‘by eye’ data monitoring. Further, an investment in developing robust data validations at the outset can be realised in subsequent trials where metadata can be simply and reliably replicated.

## Conclusions

Here we have demonstrated that the use of portable and real-time technologies at the researcher-participant interface in a multicentre complex intervention trial is a viable and efficient method for improving trial management and data collection procedures. There is scope to extend the system further by adding other functional modules to aid in other aspects of trial management. This includes schedule planning, automatic compliance, safety reporting and dissemination of trial documentation updates. Although assessing the validity of such a system in a larger-scale trial is still required, the success of this system in ENGAGE-HD has provided a basis for an accessible, secure platform for the refinement and delivery of complex intervention trials.

## Abbreviations

CRF: Case Report Form
eSource: Electronic source CRF
GCP: Good Clinical Practice
HD: Huntington’s disease
SOP: Standard operating procedures
SQL: Structured query language
